# Elevated Intraocular Pressure and Glaucomatous Optic Neuropathy: Genes to Disease Mechanisms, Therapeutic Drugs, and Gene Therapies

**DOI:** 10.3390/ph16060870

**Published:** 2023-06-12

**Authors:** Najam A. Sharif

**Affiliations:** 1 Eye-APC Duke-NUS Medical School, Singapore 169857, Singapore; gmsnas@nus.edu.sg; 2 Singapore Eye Research Institute, Singapore 169856, Singapore; 3 Department of Pharmacology and Neuroscience, University of North Texas Health Sciences Center, Fort Worth, TX 76107, USA; 4 Department of Pharmacy Sciences, Creighton University, Omaha, NE 68178, USA; 5 Department of Pharmaceutical Sciences, College of Pharmacy and Health Sciences, Texas Southern University, Houston, TX 77004, USA; 6 Imperial College of Science and Technology, St. Mary’s Campus, London W2 1PG, UK; 7 Institute of Ophthalmology, University College London, London WC1E 6BT, UK

**Keywords:** primary open-angle glaucoma, genome-wide associated studies (GWAS), ocular hypertension, optogenetics, CRISPR-Cas9

## Abstract

This review article focuses on the pathogenesis of and genetic defects linked with chronic ocular hypertension (cOHT) and glaucoma. The latter ocular disease constitutes a group of ocular degenerative diseases whose hallmark features are damage to the optic nerve, apoptotic demise of retinal ganglion cells, disturbances within the brain regions involved in visual perception and considerable visual impairment that can lead to blindness. Even though a number of pharmaceuticals, surgical and device-based treatments already exist addressing cOHT associated with the most prevalent of the glaucoma types, primary open-angle glaucoma (POAG), they can be improved upon in terms of superior efficacy with reduced side-effects and with longer duration of activity. The linkage of disease pathology to certain genes via genome-wide associated studies are illuminating new approaches to finding novel treatment options for the aforementioned ocular disorders. Gene replacement, gene editing via CRISPR-Cas9, and the use of optogenetic technologies may replace traditional drug-based therapies and/or they may augment existing therapeutics for the treatment of cOHT and POAG in the future.

## 1. Introduction

The eye is a highly specialized organ dedicated to relaying sensory information about our surroundings to the brain, which orchestrates our reactions to the visual cues and the images perceived. Consequently, vision is critical for our survival and good quality of life since our ability to read, learn, socialize, and perform daily tasks are highly eyesight-dependent [1,2]. Unfortunately, as with other organs, the eye is prone to various dysfunctions and diseases such as ocular hypertension (OHT), glaucoma, age-related macular degeneration, dry eye, diabetic retinopathy and many inherited retinal diseases (IRDs) [1,2,3,4,5,6,7,8,9]. The current review aims to describe the etiology of chronic OHT (cOHT) and one specific type of glaucoma, primary open-angle glaucoma (POAG), which is mainly caused by chronically elevated intraocular pressure (IOP) or OHT [3,4,5,6,7]. This will be followed by a high-level description of some of the most prevalent and highly associated genetic defects and variants [10] that have been linked with OHT, POAG, and patients’ responsiveness to various forms of current drug treatments [11,12,13]. Additional information on the basis of gene therapy and some key examples of its application for the treatment of OHT, POAG, and optic nerve preservation/protection will be discussed [14]. Since many patients’ OHT becomes recalcitrant to pharmaceutical therapeutics, and also since POAG patients’ glaucomatous optic neuropathy (GON) and vision continue to deteriorate although maximal medical therapy, suitable neuroprotective therapies need to be considered to protect retinal ganglion cells (RGCs), their axons and their terminal boutons within the visual centers of the brain [3,4,5,6,7]. This aspect will also be addressed relative to potential gene therapies and neuroprotective agents and treatments [7,14].

## 2. Pathogenesis of cOHT and PAOG

As mentioned above, it is deemed necessary to first outline the disease processes and factors involved at the cellular, molecular and genetic levels in the pathogenesis of cOHT and POAG for the readership of this review and this journal. Furthermore, before dealing with the role of genetic loci, genetic mutations and the nature of the gene products which are affected in the disease processes associated with cOHT and glaucoma, a brief outline of the eye anatomy and major contributors to the pathogenesis of glaucoma is necessary.

**Figure 1:**
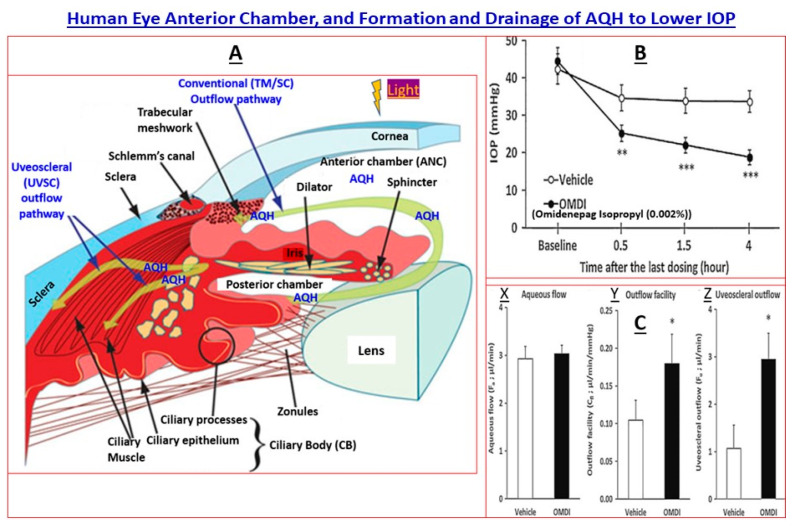
High-level anatomy of the human eye anterior chamber and the pathways involved in aqueous humor drainage to reduce IOP are shown in (**A**). (**B**) illustrates the IOP-lowering effects of a topical ocularly delivered novel non-prostaglandin EP2-receptor agonist, omidenepag isopropyl ester (OMDI), in ocular hypertensive Cynomolgus monkey eyes. (**C**) shows the promotion of AQH outflow by OMDI via both the conventional (Y) and uveoscleral (Z) routes without influencing the inflow of AQH (X) in the latter monkey’s eyes. All figures are adapted from [15,16] under open-access terms and from author’s own publications. The statistical significance is shown amongst the different groups. * *p* < 0.05, ** *p* < 0.01; *** *p* < 0.001.

Glaucomas are a constellation of heterogeneous and complex disorders that either involve only the back of the eye (BOTE) tissues (retina, optic nerve and brain connections) and/or involve front of the eye (FOTE) tissues (trabecular meshwork [TM], Schlemm’s canal [SC], ciliary epithelium [CE] and ciliary muscle [CM]) as well as the BOTE components. As illustrated in Figure 1, the eye is a complex but exquisitely designed organ composed of highly specialized cells for picturing the environment around us. At a high level, light enters the eye and passes through the cornea, aqueous humor (AQH) and pupil. As it hits the lens, it is focused onto the light-sensitive retinal cells to initiate the conversion of the light wave energy to chemical and then electrical signaling to the brain via the optic nerve. From the thalamic regions of the brain, electrical signals are sent to the neuron of the visual cortex for decoding and light/image perception. All this occurs in milliseconds. Therefore, defects, dysfunction and/or dysregulation of cellular components or the signal transduction mechanisms in the retina-optic nerve–brain network have grave consequences that cause visual impairment, which could lead to blindness. Surprisingly, in the case of acute OHT or cOHT, problems in the anterior chamber of the eye trigger neurodegeneration of the latter tissues at the BOTE and cause glaucomatous optic neuropathy (GON). In other cases, the patient’s susceptibility to detrimental factors and events in the retina/optic nerve regions directly results in GON without contributions from injurious events in the FOTE at the level of the TM and SC. Regardless, the end result of these various detrimental events and factors is loss of vision and potential blindness.

Glaucoma is characterized by irreversible visual impairment and initial loss of peripheral vision resulting from damage to the optic nerve and the demise of the associated retina ganglion cells (RGCs) and their axons, which also negatively impacts brain visual centers. Such GON due to POAG and primary angle-closure glaucoma (PACG) currently afflicts >75 million people worldwide. However, as our aging population increases and diagnosis of these glaucomas becomes easier, these numbers will approach >112 million glaucoma patients by 2040 [3,4,5]. Several forms of glaucoma exist, encompassing POAG, PACG, normotension glaucoma (NTG), and primary congenital glaucoma [3,4,5,6,7]. Secondary glaucomas include: uveitic glaucoma, traumatic glaucoma, pigmentary glaucoma, steroid-induced glaucoma, and pseudoexfoliation glaucoma (PEXG) [3,4,5,6,7]. Although many risk factors are associated with glaucomas, the major ones impacting POAG, for instance, include advanced age, family history of the disease, African heritage, chronically elevated intraocular pressure (IOP), reduced intercranial fluid pressure, poor retinal blood flow, diabetes, and to some degree systemic hypertension [3,4,7]. Of these, substantially elevated IOP beyond the normal range (10–21 mmHg) is considered a common factor linked with most forms of glaucoma, except NTG [3,4,7]. IOP is highly correlated with glaucomas based on animal models [17] and many clinical studies, e.g., [18,19,20] such that there is a 10–15% increase in the risk of a patient developing glaucoma for every 1 mmHg increase of IOP beyond normal IOP [18,19,20]. How this cOHT develops and negatively impacts the retina-optic nerve–brain connectivity structurally and functionally, and which adversely affects visual perception are incompletely understood but can presently be summed up as elevated IOP is caused by a build-up of unwanted proteins that occlude fluid drainage channels within the eye. Over time, AQH fluid accumulates in the anterior chamber of the eye and IOP rises [3,4,5,6,7,21]. If left unchecked, this compressive pressure damages the optic nerve that sends light signals to the brain and vision begins to deteriorate [3,7,22,23]. Moreover, even though conventional topical eye drop medications help reduce disease progression, about 10% of the patients become resistant to the topical therapies, thereby increasing their risk of permanent loss of vision [11,12,13]. Additional vision loss occurs due to poor compliance. A more detailed discourse about these aspects is presented below.

**Figure 2:**
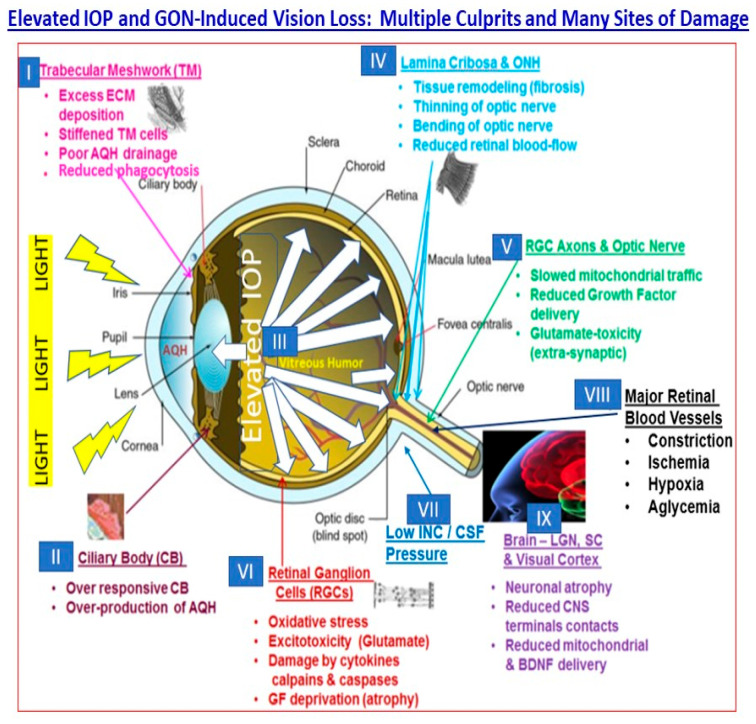
The many sites of dysfunction within the visual axis as a result of elevated IOP/ocular hypertension and glaucomatous optic neuropathy are shown in this schematic. These tissues and the cells therein, and the chemicals and factors shown, represent opportunities for intervention to mitigate the pathogenesis and progression of the cOHT, POAG and NTG. The Figure is adapted from a recent publication of the author [24] under the terms and conditions of the (Open Access) Creative Commons Attribution (CC BY) license (https://creativecommons.org/licenses/by/4.0/). Additional references include author’s own publications as in [15,16].

The balance between AQH production by the ciliary body and its drainage from the anterior chamber (ANC) of the eye is disrupted due to aging and local pathogenic and damaging factors. In brief, AQH efflux via the TM/SC into the veinous circulation is reduced due to the accumulation of unwanted excess extracellular matrix (ECM) proteins in and around the TM and due to TM cell senescence (Figure 1A and Figure 2; [21]). The other minor AQH outflow pathway, the uveoscleral (UVSC) route, is marginally impacted but becomes a major player when IOP-lowering therapies are introduced (e.g., topical ocular dosing with FP-receptor prostaglandin agonists; or with an EP2-receptor agonist (omidenepag isopropyl) that lowers IOP in OHT monkey eyes (Figure 1B) by recruiting both the TM/SC conventional outflow and UVSC outflow pathways (Figure 1C) [6,7,21]). Regardless, AQH continues to accumulate within the ANC, which raises the IOP. Under glaucomatous conditions, the TM cells are stressed, and they release the vasoconstrictor peptide (endothelin) and transforming growth factor-β2 (TGF-β2), and cytokines, and uncontrolled misfolded myocilin begins accumulating inside endoplasmic reticulum of the TM cells [3,4,7,22]. All these deleterious events cause the demise of more TM cells, and more ECM is deposited, causing a further increase in IOP, and this vicious cycle continues uncontrolled over decades, and the patient remains asymptomatic and unaware of the damage being inflicted to the retinal, optic nerve and brain cells/tissues [17,22,23].

The high IOP makes the whole globe bulge slightly with resultant mechanical distortion, disruption and damage of the delicate tissues at the BOTE, especially the optic nerve head (ONH) and the somewhat “soft” fenestrated lamina cribosa (LC) tissue of the retina. The ensuing local inflammation due to the release of cytokines, chemokines, glutamate, ATP, endothelin, and proteases, with the subsequent weakening of the LC and buckling/bending of the RGC axons and retinal blood vessels, leads to ischemia and further damage to the RGCs and their axons [3,4,5,6,7,22]. Over time, axonal transport of mitochondria and neurotrophins to and from the brain thalamic neurons and RGC cell bodies is interrupted, and the most vulnerable RGCs die; their axons wither, and atrophy and connections to the brain are lost (Figure 2). A similar fate awaits many of the neurons in the brain which serve as the relay stations (e.g., thalamic nuclei neurons) for visual information transfer from the RGCs, and for eventual visual perception (visual cortical neurons) since they are neural-activity-driven systems [3,4,5,6,7,13,23]. Peripheral vision is first to be lost, and over time, if not treated, the person can lose all eyesight in the affected eye. All the aforementioned factors and events play out over many years, and the glaucoma patient remains oblivious to the damage occurring in their eyes, optic nerve and brain until nearly half of their original RGCs and their axons are destroyed by GON and periphery-to-center vision loss continues to progress. It is at this time the patient needs to be rapidly treated with IOP-lowering medications and/or undergo surgery to reduce the elevated IOP and start preserving the remaining structure and functions of the visual apparatus [3,4,5,6,7,22,23]. 

**Figure 3:**
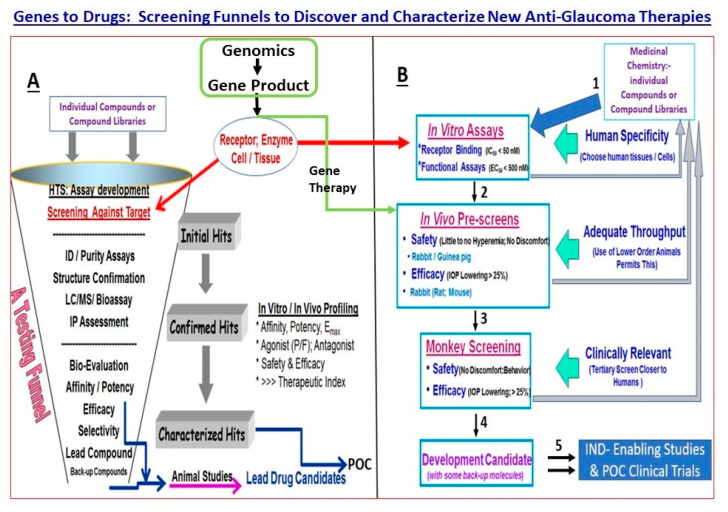
Displayed in this figure are a typical testing funnel and the components therein for synthesis of test compounds and/or gene therapy capsids and their characterization, followed by their evaluation in vitro and in vivo to eventually produce suitable compounds for progression towards Investigational New Drug (IND)-enabling studies (**A**). A more comprehensive screening paradigm for discovering and analyzing the pharmacological and biochemical features of test agents in a variety of in vitro assay systems, followed by screening in animal models of increasing complexity, is shown in (**B**). Examples of certain stage-gate passing criteria for progression down the testing funnel are also included for illustration purposes. Once the preferred compound/gene therapy has met all the prescribed criteria, it can undergo IND-enabling studies and then into proof-of-concept clinical trials for the target disease (e.g., for elevated IOP; slowing RGC/optic nerve damage). Figures are adapted from [15,16], the author’s own recent publications.

As described above, POAG results from several factors and events at many sites within the visual axis, beginning with the ANC, retina, optic nerve head, optic nerve, brain thalamic and visual cortical regions (Figure 2) [23]. Consequently, there exists a plethora of endogenous ligands, receptors, enzymes, transporters, ion channels and structural elements and their constituents whose defects or dysfunction may contribute towards the disease initiation, progression and amplification. The difficulty lies in delineating whether the differentiating factors detected in the diseased tissues, cells, or fluid biopsies compared to samples from age-matched control subjects are causative or a consequence of the disease. However, although these issues make POAG a difficult ocular disease to diagnose and treat, they also present several opportunities to intervene at multiple loci in the disease process to help retard the progression of the visual impairment and thus help preserve the eyesight of the affected patients. Some of the factors and other elements shown in Figure 2 are amenable to well-established methods to help discover and develop drugs, including gene therapy (Figure 3) [7,13,14], as investigated in animal models of eye disorders and in some cases in human subjects with varying degrees of success [7,8,9,14]. 

**Figure 4:**
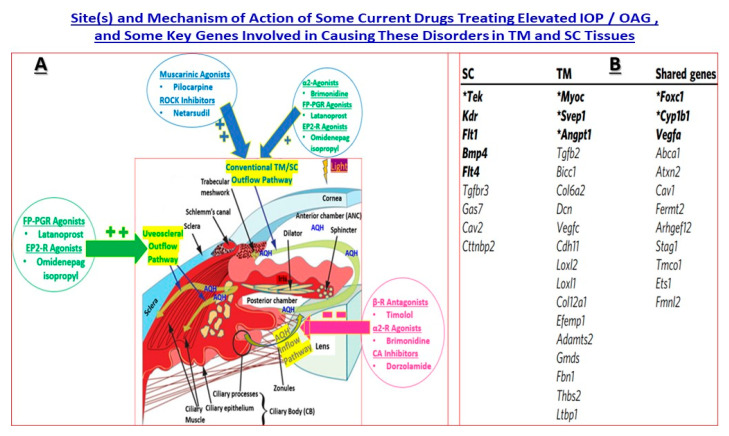
The varied mechanisms of action and pathways engagement by various drugs to promote egress of AQH from the anterior chamber of the eye via the conventional TM/SC pathway and/or via the uveoscleral pathway to relieve the elevated IOP are depicted in Figure 4A. Moreover, drugs that inhibit the production of AQH (inflow inhibitors) are also shown in (**A**). (**B**) displays a list of genes that are impacted and/or are implicated in the etiology of glaucoma (POAG/cOHT) via TM, SC and/or both tissues. (**A**) is modified from Figure 1A, which was adapted from Refs. 99 and 100 (the author’s own recent publications). The small tabulate list shown in (**B**) is derived with gratitude from [25] under Creative Commons Attribution 4.0 International License and which is a Nature Communications article.

Since cOHT is a major causative factor for POAG and many other types of glaucoma, reducing increased IOP has become the mainstay treatment to manage the disease. Several topical ocularly delivered pharmaceuticals are able to lower and control IOP with FP-class prostaglandins (e.g., Xalatan (latanoprost), Travatan (travoprost) and Taflutan (tafluprost)) being the first-line therapeutics that primarily enhance AQH outflow via the UVSC pathway but also promote TM/SC conventional outflow to some degree [3,4,5,6,7]. These and other types of approved and marketed drugs that lower IOP and their mechanisms of action (AQH inflow inhibitors or promoters of conventional or UVSC outflow of AQH) are illustrated in Figure 4, and further information on many investigational drugs active in animal models of glaucoma, and in some cases in human subjects, have been recently reported and discussed [4,6,7,21]. Surgical and microshunt device implantation techniques are utilized in patients who are poor or low responders to the medications and in those patients where even maximal medical therapy fails to lower and control IOP [4,5,6,7,9,10,11,12]. Moreover, due to ocular and systemic side effects caused by many ocular hypotensive drugs, which lead to patient incompliance, better drugs with fewer or even more reduced side effects are needed. Furthermore, despite the availability and clinical utility of the aforementioned treatments, most glaucoma patients continue to progressively lose vision and are destined to go blind. Hence, there continues a need to find new agents, devices and other treatment modalities, such as gene and cell therapies, to treat glaucomatous optic neuropathy (GON) associated with glaucoma. Regarding gene therapies, it is important to first describe the various genes and variants that have been identified and strongly associated with the pathogenesis of elevated IOP and POAG, e.g., [9,10]. 

## 3. Some Examples of GWAS for Different Forms of Glaucoma—Translational Research

Due to the enormity of the genes that have been linked thus far to OHT and POAG (e.g., [10,11,12]), it is impossible to cover the field completely in this review article. Instead, only selected areas and genes will be discussed. Thus, early studies relied on mapping genes amongst families and helped discover a prominent gene causing POAG [26,27]. Analyses of sequence-tagged site (STS) content and haplotype sharing between families affected with chromosome 1q-linked open-angle glaucoma (GLC1A) were used to prioritize candidate genes for mutation screening. A gene encoding a trabecular meshwork protein (TIGR) mapped to the narrowest disease interval by STS content and radiation hybrid mapping. This was the mutant myocilin gene, MYOC [26,27]. Since then, the techniques to find defective genes have significantly improved and now deploy GWAS, a genome-wide set of genetic variants in different individuals to see if any variant is associated with a given trait. GWAS typically focuses on associations between SNPs and traits of major human diseases but can equally be applied to any other genetic variants and any other organisms. When applied to human data, GWAS compares the DNA of participants having varying phenotypes for a particular trait or disease. These participants may be people with a disease and similar people without the disease, or they may be people with different phenotypes for a particular trait, for example, blood pressure. This approach is known as phenotype-first, in which the participants are classified first by their clinical manifestation(s), as opposed to genotype-first. Each person gives a sample of DNA, from which millions of genetic variants are read using SNP arrays. If one type of variant (one allele) is more frequent in people with the disease, the variant is said to be associated with the disease. The associated SNPs are then considered to mark a region of the human genome that may influence the risk of that disease. GWAS investigates the entire genome, in contrast to methods that specifically test a small number of pre-specified genetic regions. 

Multiple genetic studies using GWAS have helped identify and/or confirm some of the culprits associated with different forms of glaucoma. The majority of the studies have concentrated on POAG [28,29,30,31,32,33,34,35,36,37]. GWAS have also been reported for PACG [38,39,40], PEXG [41,42,43,44], NTG [45] and pigmentary glaucoma [46]. The above and even more recent GWAS [47,48,49,50,51] clearly indicate the heterogeneous nature of all forms of glaucoma and that multifactorial etiologies prevail. Importantly, for POAG, for instance, researchers were able to link POAG to defects in myocilin [26,27,37], to retinal nerve fiber disease [50], *Cacna2d1* regulation in elevated IOP [20,30,31,35,51,52], defects in the proteome of human scleral tissue [53], and that of the central cornea [31,34,40,54]. In order to seek genes that may influence glaucoma and OHT, three gene categories linked to IOP have been interrogated. These include (1) glaucoma-associated genes with mutations; (2) genes whose expression is changed in GON and (3) genes known or implicated in pathway networks that influence IOP. Accordingly, several rare variants of genes that are correlated with IOP pathogenesis have been identified and some characterized (e.g., *ABCA1*, *ADAMTS8*, *ADAMTS17*, *AFAP1*, *ANGPT1*, *ANTXR1*, *ARHGEF12*, *ARID5B*, *ATXN2*, *BICC1*, *CACNA2D1*, *CAV1-CAV2*, *CDKN2B-AS1*, *CELF1*, *CYP26A1-MYOF*, *FAM125B*, *FNDC3B*, *FOXC1*, *FOXP1*, *GAS7*, *GLCCI1-ICA1*, *GLIS3*, *GMDS*, *HIVEP3*, *INCA1*, *LMX1B*, *LOC171391*, *MADD*, *MIR548F3*, *MYBPC3*, *RPLP2-PNPLA2*, *SIX1/SIX6*, *SEPT9*, *SP11*, *TFEC-TES* and *TMCO1*) [20,27,28,29,30,31,32,33,34,35,36,47,48,49,55]. Whether any of these genes represents a “master gene” governing the etiology of OHT/POAG remains to be determined. 

## 4. Basics of Gene Therapy for Eye Diseases

The genetic basis of several human diseases is well accepted now, and amongst them are many ocular disorders [8,9,10,11,12,13,14]. Thus, eye diseases such as glaucoma, retinitis pigmentosa, age-related macular degeneration, and Type-2 Leber congenital amaurosis (LCA-2) have some documented linkage to genetic aberrations. The greatest ocular gene therapy success thus far has been in the clinical management of LCA-2, an autosomal recessive disease that has been linked to mutations in the GUCY2D, CEP290 and RPE65 genes. RPE65 is a critical enzyme involved in phototransduction and is located in the retinal pigmented epithelium (RPE) and in rods and cones of the retina. Due to RPE65 mutations, the opsins cannot capture light or transduce it into electrical responses to initiate vision. In order to overcome this problem in LCA-2 patients, voretigene neparvovec-rzyl (Luxturna^®^, Spark Therapeutics) used an adeno-associated virus-2 (AAV2)-based vector that delivered the normal RPE65 gene via a subretinal injection to replace the defective gene [8]. This procedure and therapy allowed the LCA-2 patients to regain some of their eyesight. 

Even though a defective gene could be repaired by the insertion of a functional gene to produce a missing enzyme in LCA-2 patients, it is becoming clear that genetic anomalies can also adversely impact the availability of endogenous ligands, ion channels, enzymes, receptors and/or their transmembrane effector components which alter patient responsiveness to and/or metabolism of natural ligands or drugs prescribed for certain ocular disorders [11,12,13]. The advent and utility of novel techniques, including genome-wide associated studies (GWAS), high-density single nucleotide polymorphism (SNP) microarrays, and next-generation sequencing, has rendered genetic associations with many eye diseases possible [10,28,29,30,31,32,33,34,35,36,37,38,39,40,41,42,43,44,45,46,47,48,49,50,51,52,53,54,55]. Thus, identification of GWAS-identified associations, disease-genomic regions, target protein(s) and potential biological pathways implicating the latter can be identified and incorporated into appropriate drug discovery and development campaigns [4,6,7]. The identification of genetic loci, alleles and variants/sub-variants related to the disease phenotype can then lead to more specific therapies with a lower propensity to cause damaging side effects, and thus these therapeutics become more patient-centric and can become personalized medicines. Although gene therapies may result from such discoveries [8,14,22], it is quite feasible that the target ligand or protein may be amenable to classical pharmacological treatment, which can be found by empirical means or through target-directed screening techniques and technologies, including high-throughput screening coupled with high-content phenotypical screening [4,6,7]. After in-depth non-clinical and clinical evaluations, the new therapy may be approved by a health agency to mitigate the signs and/or symptoms of the disease being targeted. Whilst translating genomic information into drugs or other forms of medications is a lengthy and expensive process, with a high rate of attrition, patients with eye disorders await novel treatment modalities [1,2,3,4,5,6,7,8,9,10]. Moreover, although the attractiveness of gene therapy is that correction of a genetic defect may cure rather than just treat the symptoms or signs of the disease, this is no easy task since liver toxicity, oncogenic issues and blood disorders plague this form of treatment for systemic diseases. However, as mentioned above, the only health agency-approved ocular gene therapy, Luxturna (voretigene neparvovec; AAV2 vector containing human RPE65 cDNA), to treat LCA-2 is relatively safe due to its local delivery into the eye [8,9]. As such, the easy accessibility of gene therapy delivery to the ocular tissues provides a unique opportunity to directly inject the viral vectors and their cargos and, through appropriate tropism, target specific cells and retain the capsids within the eye without any appreciable leakage to the systemic circulation. 

Today, many types of viral vectors are used to deliver the target gene(s), including AAV2, AAV6, AAV8 and lentivirus vectors [8,14,32]. The AAV vectors are deployed where the goal is a chronic expression of the gene product and where the viral DNA does not integrate into the host genome, and thus, the risk of mutagenesis is minimal. AAV vectors carry genes with good fidelity, and in some cases, there is less concern about off-target integration [8,14,32]. Insertional oncogenesis is a risk associated with AAV-based vectors, but different serotypes permit the minimization of patient rejection and off-target effects. Lentiviral vectors are deployed in ex-vivo cell therapies and can transduce nondividing cells incorporating a larger transgene than that possible with AAV vectors [8,14,32]. On the horizon are non-viral vectors that include lipid nanoparticles that can deliver genes to appropriate tissues. These approaches are safe, but the gene transduction level may be limited. Finally, in terms of routes of delivery, if the disease being targeted is related to the anterior chamber tissues, then the AAV-gene complex is delivered by injection into the AQH (intracamerally), and cells within the CM, TM, SC or ciliary body can be effectively transduced. However, if the cells/tissues at the back of the eye need to be involved, then either intravitreal, suprachoroidal or sub-retinal routes of administration of the capsid(s) need to be performed. Additional factors to consider have been recently reviewed [8,14,32].

## 5. Drug Discovery and Development for cOHT/POAG—Genes and Pathway Analysis

For brevity and conciseness, I only intend to focus on some of the major genes and signaling pathways that have been found to be strongly associated with elevated IOP (OHT), POAG and/or NTG. Some of these are already bearing fruit in terms of drug discovery target identification and linkage of certain compounds to the latter with efficacy in lowering IOP, for instance. 

### 5.1. MYOC Gene and Mutant Myocilin Raises IOP

*MYOC* encodes the myocilin protein and is also known as a TM-glucocorticoid response (TIGR), GLC1A, or TIGR/myocilin gene, being the first gene linked to the pathogenesis of POAG [26,27,37,56]. Myocilin is a secretory protein released under normal circumstances by the TM cells. However, it is present in TM and SC cells, the sclera, the ciliary body, the retina and in the optic nerve. Myocilin expression is regulated by several agents (e.g., steroids, TGF-β, and optineurin) and by oxidative and mechanical stress [26,27,37,56]. Intracellularly, myocilin is located in the endoplasmic reticulum (ER), the Golgi apparatus, and mitochondria, whereas cytosolically, it is found in exosomal vesicles associated with microtubules. Normally folded and secreted, myocilin has beneficial structural and functional roles in regulating AQH dynamics within the ANC of the eye and is thus important for regulating IOP. Normally, ~40% of myocilin undergoes proteolytic cleavage in the ER, yielding a 35 kDa C-terminal olfactomedin domain fragment and a 20 kDa fragment comprising the N-terminal coiled-coil domain [27,37,56]. The olfactomedin domain is co-secreted with unprocessed full-length myocilin, while the N-terminal domain is maintained intracellularly inside the ER. The olfactomedin domain is the site of more than 90% of all disease-causing myocilin variations, which lead to the misfolding of myocilin, its aggregation, and accumulation within cells [1,37,56]. Such mutant myocilin is toxic to TM cells which causes them to malfunction and which kills many of them [56,57]. Consequently, AQH accumulates and raises IOP, which then damages the RGC/optic nerve–brain sites described above, leading to visual impairment and loss of peripheral vision [3,4,5,6,7,27,37,56]. 

**Figure 5:**
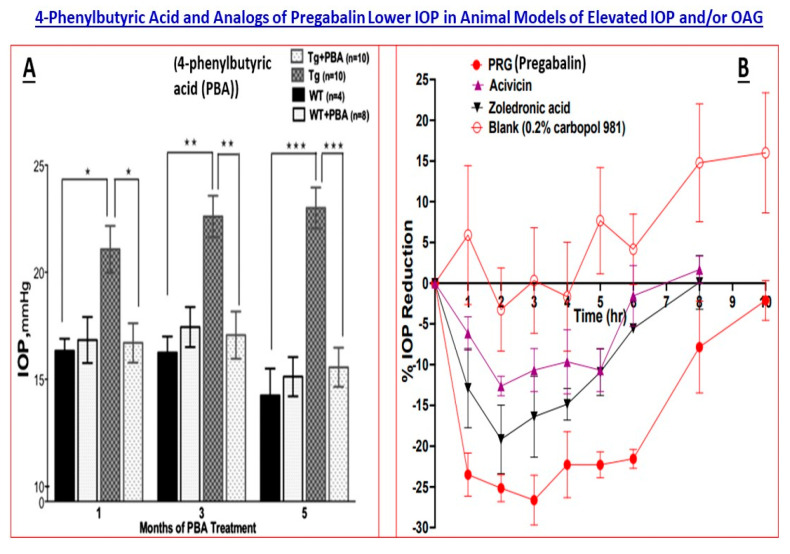
The 4-phenylbutyric acid (PBA)-induced ocular hypotension in a mouse model of myocilin-induced glaucoma (transgenic mice) is shown in (**A**) [57]. In the adjoining (**B**), the time-dependent IOP-lowering action of pregabalin and its analogs (CACNA2D1 inhibitors) is displayed [59]. Both Figures are adapted from the aforementioned references under the terms and conditions of the (Open Access) Creative Commons Attribution (CC BY) license (https://creativecommons.org/licenses/by/4.0/). The statistical significance is shown amongst the different groups. * *p* < 0.05, ** *p* < 0.01; *** *p* < 0.001.

From a myocilin-induced glaucoma phenotype treatment perspective, topical ocular sodium 4-phenylbutyrate (4-PB) significantly reduced myocilin accumulation and enhanced its secretion, and reduced ER-stress and lowered IOP in *Tg-MYOC* (*Y437H*) mice [57] (Figure 5A). Similarly, the knockdown of Grp94 (a heat-shock chaperone protein) with siRNA or inhibition of Grp94 by pharmacologic means caused the degradation of toxic mutant myocilin via autophagy and helped rescue cells from mutant myocilin toxicity [58]. Whether such protein chaperone inhibition can modulate IOP in vivo remains to be determined, but relieving ER stress goes a long way to providing cellular protection of not just TM cells but also RGCs, thus imparting neuroprotection. Nevertheless, the above-mentioned studies illustrate how GWAS led to investigations for pharmacotherapy for mutant-myocilin-induced glaucoma and which may yield important drugs for this form of glaucoma.

In a similar vein of finding novel treatments for ER-related stress reducers and thus indirectly treating POAG/NTG, a very recent study is noteworthy [48]. ER is a dynamic organelle involved in protein synthesis, transport and folding, lipid and steroid synthesis, carbohydrate metabolism and calcium storage and is critical for cellular homeostasis. ER stress activates protein kinase RNA-like endoplasmic reticulum kinase (PERK)-mediated unfolded protein response (UPR) signaling pathway. PERK pathway can also be modulated by carbon monoxide, which is implicated in POAG. Furthermore, ER stress-mediated induction of the UPR is directly correlated with activation of the three ER transmembrane sensor proteins such as PERK, inositol requiring enzyme-1 and activating transcription factor 6 (ATF6). ER homeostasis is maintained by ER chaperones such as glucose-regulated protein 78 (GRP78). Thus, it was significant that LDN-0060609, a small molecule PERK inhibitor, provided a decrease of ER stress marker proteins within human TM cells. This agent also effectively increased the viability and cell proliferation and reduced DNA damage and apoptosis, and restored the normal morphology of human TM cells subjected to ER stress conditions, thereby rescuing and preserving the phenotype and functional properties of the cells [48]. 

### 5.2. Cacna2d1 Gene and Linkage to a Ca^2+^-Channel 

*Cacna2d1* gene was recently identified and linked to elevated IOP and POAG using systems genetics, an extension of complex trait analysis that examines large sets of genotypes and phenotypes to investigate the genetic basis of disease traits [52]. *Cacna2d1* encodes a preproprotein which is cleaved into many chains that form the alpha-2 and delta subunits of the voltage-dependent Ca^2+^-channel complex. CACNA2D1 is a glycosylphosphatidylinositol-anchored subunit typically associated with the caveolin Cavα1 pore within L-type Ca^2+^-channels. The *CACNA2D1* gene is highly polymorphic, with several hundred SNPs and 30 insertions/deletions, and the human version has several splice variants. CACNA2D1 is expressed in CB, ciliary muscle, TM and pregabalin, a drug used for neuropathic pain treatment that exhibits a high affinity for this Ca^2+^-channel complex. Furthermore, pregabalin and some other novel CACNA2D1-inhibitors administered topical ocularly in mice lowered IOP up to 30% from the baseline over a few hours [52,60] (Figure 5B). Whether a new generation gabapentinoid drug, mirogabalin [59], can reduce IOP to the same or greater level as pregabalin and with a longer duration of action remains to be determined. The aforementioned studies illustrate how genome-based investigations can define the roles of certain proteins in normal/abnormal functions related to glaucoma and can lead to the discovery of novel treatments for elevated IOP/POAG exemplified by the CACNA2D1-inhibitors [52,59,60]. 

### 5.3. CAV1/CAV2 Genes and Caveolin Proteins

GWAS on Icelandic, European and East Asian individuals showed that variant rs4236601 in *CAV1* and *CAV2* on chromosome 7q31 has a significant influence on POAG pathogenesis [61]. Another common SNP rs3801994 at the *CAV1/CAV2* locus in Chinese and Japanese individuals was linked to POAG and NTG in certain Chinese people [39,40,61]. *CAV1/CAV2* genes encode caveolin proteins which are important for signal transduction, vesicular transport, and cholesterol homeostasis, and which are enriched in human TM/SC cells, ciliary muscle, and retina and which interact with L-type Ca^2+^-channels (see above). Caveolins modulate endothelial nitric oxide synthase functionality and affect vascular tone and TM relaxation such that *CAV1*-knockout mice display elevated IOPs [62,63,64]. The latter mice express very little caveolae (special lipid rafts) in their TM/SC cells. Delivery of short hairpin shRNAs for *CAV1/CAV2* inserted into lentiviral vectors into TM cells of mice resulted in increased TM/SC outflow facility for CAV-1 and decreased facility for CAV-2 [62], indicating a complex interplay between these proteins. However, elevating the expression and activity of CAV1/CAV2 proteins and/or delivering caveolae could be useful in treating POAG and NTG. Indeed, a very recent report showed that intravitreal injection of acteoside, phenylpropanoid glycoside, reduced RGC loss and oxidative stress by upregulating CAV1 [63]. 

### 5.4. TMCO1 Gene and Its Protein

A variant, rs4656461, near TMCO1 on chromosome 1q24 was associated with severe POAG-mediated visual field loss in a GWAS of a Caucasian cohort [65]. This variant was also correlated with POAG pathogenesis in a Pakistani population, and rs4656461 and rs7555523 variants at TMCO1 showed significant association with POAG in the Chinese population since carriers of these risk alleles at TMCO1 seemed to be predisposed to the development of OHT and POAG [65,66]. *TMCO1* gene product is a transmembrane protein with a coiled-coil domain that is located in the Golgi apparatus, ER and mitochondria. The protein is highly enriched in the ciliary body, TM, lamina cribrosa, optic nerve, and retina (especially RGCs). It regulates Ca^2+^ homeostasis in the ER, regulates the cell cycle, and is implicated in apoptotic cell death. Interestingly, TMCO1 seems to interact with the *CAV1* gene, thus implicating the associated signaling pathways of the CACNA2D1 protein. Consequently, agents or treatment paradigms that modulate the expression of TMCO1 and its encoded protein may have utility in addressing OHT/POAG.

### 5.5. GAS7 Gene and Its Protein

GWAS helped identify rs11656696 polymorphism located in *GAS7* and showed its association with IOP level in human subjects from population-based investigations from the Netherlands, United Kingdom, Australia, Canada and other regions. The analyses showed that the rs11656696 polymorphism is directly linked with glaucoma pathogenesis. Another recent study demonstrated that rs11656696 polymorphism in *GAS7* is directly correlated with POAG pathogenesis and may constitute a protective factor against POAG in a Chinese population [67]. However, polymorphism rs11656696 is not associated with IOP and is not considered a risk factor for POAG in the Saudi Arabian population. These region-specific alterations illustrate the diversity of effects of SNPs on the glaucoma phenotypic relationships. *GAS7* belongs to the Pombe Cdc 15 homology family, and its gene product regulates microfilament organization, neuronal differentiation, apoptosis, tyrosine kinase receptor activity, and control of the cell cycle progression. GAS7 protein is highly expressed in the TM, with moderate levels in the ciliary body, amacrine retinal cells, lamina cribrosa and optic nerve tissues all connected with IOP regulation and visual information transmission and processing. Additionally, *GAS7* appears to interact with other genes implicated in glaucoma pathogenesis, such as *MYOC*, *OPTN*, *WDR36*, *CAV1*, nitric oxide synthase 2 (*NOS2*), forkhead box C1 (*FOXC1*), apolipoprotein E (*APOE*), amyloid precursor protein (*APP*) and clusterin (*CLU*). Additional studies have shown that *GAS7* interacts with *MYOC* and CAV1 via β-catenin (*CTNNB1*) and RhoA (*RHOA*). Furthermore, *GAS7* is regulated by TGF-β, which is implicated in trabecular outflow and optic disc development. Taken together, *GAS7* and its protein product appear central to regulatory processes and activities connected with normal and abnormal functions of genes connected to the pathology of POAG/NTG. Hence, drugs and antibodies that can modulate the GAS7 gene and GAS protein expression and function will have a major influence on deciphering their exact function(s) and would lead to a new generation of treatment modalities for POAG/NTG.

### 5.6. ABCA1 Gene and Its Transporter Protein

*ABCA1* gene product is a transporter protein that mediates the efflux of cholesterol and phospholipids between the Golgi apparatus and cell membrane. It is expressed in TM/SC cells, optic nerve, and retina, with greatly up-regulated presence in the TM cells from POAG patients. ABCA1 is known to co-localize and interact with Cav1 through its scaffolding domain, and this interaction induces the oligomerization of Cav1 and its exit from the Golgi network. Recent studies have highlighted the regulation of AQH outflow via the caveolin-1/endothelial NO synthase/NO pathway by ABCA1, which is different from its traditional role in mediating cholesterol efflux [64]. An agonist for liver X receptors (GW3965) that also activates ABCA1 significantly increased conventional outflow facility following intracameral administration and lowered IOP in mice [68]. Furthermore, overexpression of the ABCA1 gene protected against RGC apoptosis by partially blocking annexin-A1 nuclear translocation and prevention of inflammation [68]. Such studies clearly demonstrate that GWAS-identified genes linked to POAG can be utilized for drug discovery and development to manage the disease process, at least in animal models. Translation of such findings to the human conditions awaits further ratification and more comprehensive investigations. 

### 5.7. ANGPT1 Gene and Its Angiopoitin-1 Protein

*ANGPT1* gene encodes angiopoitin-1, which is an important protein for blood vessel development and which is linked to signaling via tyrosine kinase with immunoglobulin-like and epithelial growth factor-like domains-2 (Tie-2). Since SC cells and overall SC structure are of lymphatic origin, defects in the *ANGPT1* gene cause AQH drainage issues and lead to elevated IOP [25]. This was confirmed using an antibody to ANGPT1 [69]. AKB-9778, the target for vascular endothelial protein tyrosine phosphatase (VE-PTP) and a regulator of Tie-2, lowered IOP in patients treated subcutaneously for diabetic eye disease. Furthermore, AKB-9778 dose-dependently lowered IOP in rabbit and mouse eyes by enhancing TM/SC AQH outflow. Mechanistically, AKB-9778 activated Tie-2 and increased the filtration area of SC for AQH drainage in wild-type and Tie2^+/−^ mice [70]. These studies provided a novel target for addressing OHT and glaucoma with a lead compound directed at an endogenous enzyme (VE-PTP).

### 5.8. CDKN2BAS Gene and Its Cyclin Proteins

*CDKN2BAS*, also known as an antisense non-coding RNA, is strongly associated with POAG and is significantly upregulated in the retina of a rat model of glaucoma. *CDKN2BAS1* SNPs rs1063192 appear to be protective against POAG in the Afro-Caribbean population of Barbados and in Europeans [65]. Others have suggested that rs1063912 constitutes a common protective variant for POAG in Africans, African Americans and Indians [6]. *CDKN2BAS* is involved in the regulation of the expression of *CDKN2A* and *CDKN2B,* which encode cyclin-dependent kinase inhibitors. Both of the latter inhibitors of kinases are crucially involved in regulating cellular proliferation and blocking cell-cycle progression and apoptosis and influencing stem-cell self-renewal. Hence, *CDKN2BAS* polymorphisms may contribute to RGC apoptosis and subsequently to glaucoma development. CDKN2B is implicated in the TGF-β signaling pathway and thus would be involved in ECM protein deposition/regulation affecting TM/SC cell functionality and ONH structural integrity, both elements being important in POAG/NTG pathology. Thus, modulation of CDKN2A/2B directly through pharmacological treatment and/or through manipulation of the *CDKN2BAS* gene may be useful therapeutically for POAG/NTG. Indeed, antisense oligonucleotide to TGF-β2, injected intravitreally in POAG patients undergoing trabeculectomy, enabled a reduction in IOP persistently <10 mmHg over the three-month postoperative observation period [71,72].

### 5.9. SIX1/SIX6 Gene and Its Protein

Both *SIX1* and *SIX6* genes are implicated in congenital glaucoma, and an SNP rs10483727 located in *SIX1/SIX6* is linked with POAG [73]. Two SNPs, rs10483727 and rs33912345, are significantly correlated with NTG and POAG, especially with an increased incidence risk of NTG in the Chinese population. Additionally, rs10483727 was directly connected with a decrease in the global and different sectoral retinal nerve fiber layer (RNFL) thickness and reduced RGC number in individuals of European descent. Even though the functional role of the SIX1/SIX6 protein is unknown, some connectivity to the regulation of cell development, proliferation, differentiation, survival, and migration have been elucidated. *SIX6* gene is expressed in the ganglion cell layer and inner nuclear layer of the retina and in human optic nerve and visual brain centers. Modulation of this gene and its encoded protein represent targets for POAG/NTG intervention and require further study.

### 5.10. NTF4 Gene and Its Protein

Human *NTF4* gene product has a key role in the activation of tyrosine kinase B (TrkB) receptor on RGCs and inhibits their apoptotic cell death in in vitro cellular models and also in in vivo animal models of neurodegeneration. Accordingly, influencing this gene towards a gain of function would have therapeutic outcomes to rescue RGCs from different types of insults, as in POAG/NTG. Indeed, a relatively recent study showed RGC protective effects of genetically delivered brain-derived growth factor and its effector receptor protein [74]. Hence, defects in the *NTF4* gene linked to POAG/NTG can be potentially overcome by exogenous neurotrophin replacement therapy to treat these diseases.

### 5.11. OPTN Gene and Its Adapter Protein 

Optineurin (OPTN), an adaptor protein, is directly involved in the mediation of a variety of cellular processes, including vesicle trafficking, cell signaling, and autophagy. *OPTN* gene is expressed in TM, cornea, non-pigmented ciliary epithelium, iris, and retina, and OPTN protein has been found in the AQH. POAG individuals with *Glu50Lys* mutation in *OPTN* have primarily exhibited early-onset severe optic nerve damage that occurs without IOP elevation [75]. However, two *OPTN* mutations, Glu50Lys and Arg545Gln, have been identified in many studies of NTG patients. Furthermore, another variant of the *OPTN* gene, Met98Lys, has been detected more frequently in NTG patients, primarily in Asian cohorts. Surprisingly, a mutation in the OPTN-interacting protein, TANK binding protein 1 (TBK1), also can cause NTG [76]. These aspects illustrate how IOP-independent genetic defects can directly damage the visual apparatus with ensuing vision loss.

### 5.12. TBK1 Gene and Its Kinase Protein

*TBK1*, an IκB kinase (IKK)-related kinase, is associated with interferon regulatory factor (IRF)- and nuclear factor (NF)-κB-activation and is directly correlated with 1–2% cases of NTG. Additionally, as mentioned above, OPTN protein may directly interact with TBK1, which supports its role in glaucoma pathogenesis. TBK1 was upregulated in acute IOP elevation-induced ischemic retinas mouse model, and pre-treatment with the TBK1 inhibitor BX-795 reduced p16INK4a (p16) expression and RGC senescence [77] by inhibiting the inflammatory cascade [78]. Thus, this exemplifies how the genetic studies ultimately led to testing for and discovery of a suitable agent that was able to ameliorate at least some of the symptoms associated with the disease. 

## 6. Genes That Affect Patient Responsiveness to Drug Treatments 

Many new genes and variants have been discovered and reported for POAG [40,47,49,50,55], which require in-depth analysis and linkage to disease and network pathways exploration to permit future drug and/or gene therapy discovery for these blinding diseases. 

FP-prostaglandin agonists (FP-PGAs) are first-line therapeutic agents for lowering and controlling elevated IOP in OHT/POAG and NTG patients [3,4,5,6,7,79]. However, FP-prostaglandin receptors (FP-Rs) are prone to desensitization, and thus the drug concentration and dosing frequency regimen have to be carefully established through clinical trials to treat OHT/glaucoma [3,4,5,6,7,79]. Likewise, increasing evidence indicates that the heterogeneity amongst OHT/POAG/NTG patient responses to topical ocular drugs to lower IOP exists that ranges from non-responders to low responders and to full responders. Genetic and environmental factors cause such variable ocular hypotensive responses to FP-PGAAs and β-blockers [9,10,11,12,13]. Indeed, pharmacogenomic studies have identified 5 SNPs related to response to latanoprost, with FP-receptor SNPs associated with good (rs6686438, rs10786455) and no (rs3753380, rs6672484, rs11578155) responses [80,81]. Another study identified SNPs in the FP-receptor and solute carrier organic anion transporter family member 2A1 (*SLCO2A1*) genes such that 2 SNPs-rs3766355 in the FP-receptor and rs4241366 in SLCO2A1correlated with good drug response to latanoprost [82]. Poor or low responsiveness to latanoprost in some patients has also been linked to SNPs in the MMP-1 gene [5], and low expression of the transcription factor protein, FOXC1, results in the low-level generation of PG-receptors resulting in a very poor response to latanoprost [13]. Interestingly, FOXC1 mainly regulates EP3-receptor expression [11]; thus, a positive interaction between the FP- and EP3-receptors seems likely. Lastly, copy number variants located in an intronic portion of the muscle-enriched A-type lamin-interacting protein (encoded by the *MLIP* gene) appear to predict a good response to FP-PGAAs, and concomitantly a poor/low response to β-blockers like timolol in terms of IOP-lowering [11]. 

## 7. POAG and Gene Therapy

Gene therapies are increasingly being considered and used to treat animal and human disorders. In the eye field and, specifically, OHT/glaucomas, rapid progression in the development of safer and efficacious adenoviral vectors (AAV) and their deployment has led to some successes, at least in animal models of disease. Thus, intracameral injection of a glucocorticoid-inducible AVV carrying a human *MMP-1* gene lowered IOP by 70% in sheep [83], and AAV-*MMP3* gene insertion into corneal endothelial cells in vivo also produced a significant reduction of IOP [84], thereby confirming the role of MMPs in abrogating OHT as with FP-receptor agonists [79,85]. A dual gene therapy approach showed that lentiviral vectors stably expressing cyclooxygenase-2, and another carrying an FP-receptor transgene, successfully reduced IOP in cats for up to 5 months [85]. In another study, genetic silencing of beta-adrenergic receptors in the ciliary body with siRNAs to reduce AQH production lowered IOP in mice [86] and also in humans [87]. Similarly, efficacious and sustained OHT reduction in monkeys was achieved using an siRNA against the nuclear factor of kappa light polypeptide gene enhancer in B-cells inhibitor alpha, which reduces inflammation [88]. 

From a neuroprotection angle, delivery of neurotrophin genes and its receptor protein to damaged or dying RGCs in order to preserve them and reduce vision loss also appears very promising for preserving vision in glaucoma suspects and in patients suffering from POAG/NTG [74]. More recently, self-complementary AAV2 encoding a complement C3 protein, a destructive RGC attack and cell death-causing element, intravitreally delivered in rats protected the RGCs when the animals were subjected to ischemia/reperfusion-induced retinal injury [89]. Similarly, delivery of gene therapy for the X-linked inhibitor of apoptosis [90] and BCLX, the endogenous antagonist of BAX (a damaging transcription factor that causes apoptosis) [91], afforded protection and preservation of RGC cell structure and function in two different rodent models of glaucoma. A very recent report described that the over-expression of a beneficial transcription factor (MAX) in rodent models of GON imparted neuroprotective activity [92]. Finally, the use of the novel clustered regularly interspaced short palindromic repeats-Cas9 (CRISPR-Cas9) technology to edit specific genes has been accomplished in animals to lower and control IOP [93,94], but now requires to be demonstrated in human subjects in the future in terms of OHT/OAG/GON treatments. Other examples of gene therapy for IOP reduction and RGC protection are also available [32,72,74,84,85,86,87,88,89,90,91,92,93,94].

In conclusion, accounting for all the factors and network pathways, receptors, enzymes and ion channels/transporters involved in the etiology and progression of OHT/OAG/NTG described above, several mitigation strategies have been adopted and others need to be considered. It is important to consider the structural and functional elements involved or implicated in order to ameliorate the signs and symptoms of glaucomas. Unraveling the genetic basis of different forms of glaucoma and the associated SNPs that are implicated in the disease pathogenesis, with or without the involvement of elevated IOP, has clearly been useful in identifying key targets for drug discovery and development. Further progress in this endeavor using next-generation sequencing and the use of various novel treatment paradigms will continue to advance the field to help patients suffering from GON. Such treatment paradigms may include the following for current and future drug development to help combat OHT/POAG/NTG: (1). Novel target receptor agonists or antagonists; (2). Novel target enzyme activators/inhibitors; (3). Specific siRNAs to down-regulate and specific RNA-based technologies to up-regulate activity in target-specific pathways/networks; (4). AAV- and lentivirus-vectors delivering targeted genes, and use of CRISPR-Cas9 technologies; (5). Target cell-derived exosomes; (6). Target cell-derived miRNAs; (7). Chemical-sequestering, receptor- or enzyme-directed antibodies and nanobodies; (8). Nutraceutical, electroceutical and ultrasound therapies directed to the front and back of the eye, etc. [24,95,96]. As advances in GWAS and other genetic techniques add to our knowledge about genetic and epigenetic alterations affecting various elements of OHT, POAG and NTG [97,98], more targets for therapeutics discovery and development will become available. Indeed gene product networks and gene interactions powerfully impact disease initiation and progression, and finding suitable loci and nodes for therapeutic intervention will always present new opportunities and challenges. Using such bioinformatic nodes and network analyses information, as has been deployed for dry AMD and IRDs [99,100] to unearth novel and repositioning of existing drugs, researchers are sure to make meaningful discoveries for the treatment of OHT/POAG over the next few years. Potential interactions of the several genes discussed above and their relationship to OHT have been presented in a pictorial manner [97], and novel pathways and components have been discussed [25]. These genes and gene products represent novel targets for drug discovery and gene therapy. We await progress in all these areas so that patients afflicted with eye diseases can benefit from not just traditionl small molecule drugs but also gene-based therapeutics. 

## 8. Concluding Remarks

The pathogenesis of many eye diseases can be traced to certain genetic defects at the cell nuclear level and/or due to deficiency or dysfunctions in the gene product proteins and/or their natural ligands. In other cases, the gene products and their interaction with cellular organelles, intracellular chemicals, ion channels, receptors and enzymes aberrantly alter the cellular biochemistry, thereby inducing the disease. Indeed, deleterious environmental factors, deficiencies in nutraceuticals, inflammation and overstimulation of the immune system also cause or synergize with genetic problems to exacerbate the ill health of the visual system at the structural and functional levels. Therefore, the identification and characterization of genes causing ocular diseases offer pathways toward the discovery of potentially beneficial treatment modalities.

The discourse above has clearly shown the involvement of several types of genes and their protein products to directly or indirectly influence many ocular disorders, with special reference to POAG and cOHT, and GWAS have been crucial in identifying such associations. Since eyesight is our most valued sense, it is vital that more research resources be directed at discovering novel drugs, antibodies, peptides and other treatment paradigms, such as gene- and cell therapies to treat ophthalmic diseases. Patients suffering from these eye disabilities are waiting. It is certain, however, that pharmacogenomic approaches for future drug discovery, development and health agency approvals will provide suitable solutions as technologies and treatments improve. Personalized medications will also emerge as patient genomics are better understood and specific therapies become available for prescription to target individual patients with specific genetic defects. However, the many hurdles that pervade gene therapies, including use of CRISPR-Cas9 technology [8,14,32], need to be overcome and translated from the animal models of ocular diseases into human subjects to ensure proper safety and efficacy parameters are adequately addressed. This is indeed a challenging but exciting time for novel genetic therapies being discovered, characterized and introduced into medical management of not only cOHT and POAG but many other eye disorders [14,32,94,100].

## Data Availability

Not applicable.
